# XIAP inhibitor embelin induces autophagic and apoptotic cell death in human oral squamous cell carcinoma cells

**DOI:** 10.1002/tox.22450

**Published:** 2017-07-19

**Authors:** You‐Jin Lee, Bong‐Soo Park, Hae‐Ryoun Park, Su‐Bin Yu, Hae‐Mi Kang, In‐Ryoung Kim

**Affiliations:** ^1^ Department of Oral Anatomy School of Dentistry, Pusan National University, Busandaehak‐ro, 49, Mulguem‐eup Yangsan‐si Gyeongsangnam‐do 50612 South Korea; ^2^ Department of Oral Pathology School of Dentistry, Pusan National University, Busandaehak‐ro, 49, Mulguem‐eup Yangsan‐si Gyeongsangnam‐do 50612 South Korea; ^3^ BK21 PLUS Project, School of Dentistry Pusan National University, Busandaehak‐ro, 49, Mulguem‐eup Yangsan‐si Gyeongsangnam‐do 50612 South Korea

**Keywords:** apoptosis, autophagy, embelin, oral squamous cell carcinoma, XIAP inhibitor

## Abstract

Embelin is an active ingredient of traditional herbal remedies for cancer and other diseases. Recently, it has been suggested that autophagy may play an important role in cancer therapy. However, little data are available regarding the role of autophagy in oral cancers. Therefore, we conducted this study to examine whether Embelin modulates autophagy in Ca9‐22. Our results showed that Embelin had anticancer activity against the Ca9‐22 human tongue squamous cell, and we observed that autophagic vacuoles were formed by MDC and AO. We also analyzed Embelin‐treated Ca9‐22 cells for the presence of biochemical markers and found that it directly affected the conversion of LC3‐II, the degradation of p62/SQSTM1, full‐length cleavage formation of ATG5‐ATG12 complex and Beline‐1, and caspase activation. Rescue experiments using an autophagy inhibitor showed Embelin‐induced cell death in Ca9‐22, confirming that autophagy acts as a pro‐death signal. Furthermore, Embelin exhibited anticancer activity against Ca9‐22 via both autophagy and apoptosis. These findings suggest that Embelin may potentially contribute to oral cancer treatment and provide useful information for the development of a new therapeutic agent.

## 1 INTRODUCTION

Embelin (2,5‐dihydroxy‐3‐undecyl‐1,4‐benzoquinone) is an inhibitor of X‐linked inhibitor of apoptosis protein (XIAP); it is derived from the fruit of *Embelia ribes* and has been demonstrated to possess therapeutic properties, such as anticancer, antioxidant, anti‐inflammation, antidiabetes, and antihelminthic qualities.^1^, ^2^ XIAP is the most potent member of the inhibitors of apoptosis proteins (IAP) gene family. XIAP binds and inhibits caspase and therefore inhibits cell migration and invasion and induces apoptosis.^3^ Previous studies have demonstrated the potential of Embelin as an antitumor agent to induce cell growth inhibition and apoptosis in different human cancers.^4^, ^5^, ^6^

Autophagy is an evolutional phenomenon by which long‐lived proteins and damaged organelles within cells are digested in lysosomes.^7^, ^8^ Autophagy also promotes cancer cell survival under conditions of stress and functions as a defense mechanism in response to various anticancer drugs.^9^, ^10^ Therefore, the induction of autophagic cell death by anticancer reagents has been recognized as an important component of cancer therapy.^11^, ^12^, ^13^

Oral squamous cell carcinoma (OSCC) is the most common type of oral cancer and is responsible for a substantial portion of cancer‐related deaths, affecting nearly 500 000 patients annually worldwide.^14^ OSCC is one of the most persistent malignancies and remains incurable despite aggressive therapies.^15^ Patients with OSCC are currently treated with classical treatment modalities consisting of surgery, radiotherapy, and/or chemotherapy, but OSCC still shows significant mortality rates.^16^, ^17^, ^18^ Therefore, new therapeutic approaches have been investigated, with the use of natural agents being one of the most promising anticancer treatments.

Treatment with Embelin also has been examined in the course of cancer treatment and has been shown to induce apoptosis in cancer cells. However, no reports have yet examined the effects of Embelin on autophagy in an OSCC human oral squamous carcinoma cell line. This study was conducted to investigate whether Embelin can induce autophagy in OSCC cells and to determine the underlying molecular mechanism.

## 2 MATERIALS AND METHODS

### 2.1 Reagents

The following reagents were obtained commercially: 3‐[4,5‐dimethylthiazol‐2‐yl]2,5‐diphenyl tetrazolium bromide (MTT), monodansylcadaverine (MDC), acridine orange were purchased from Sigma (St. Louis, Missouri). 3‐Methyladenine (3‐MA, class III PI3K inhibitor) was obtained from Calbiochem (La Jolla, California). Antibodies against the cleaved form of caspase‐3, caspase‐8, Beclin‐1, and PARP were purchased from Cell Signaling Technology (Beverly, MA). Antibodies against LC3 (Sigma) were also used. The p62/SQSTM1, caspase‐9, ATG5‐ATG12 complex, GAPDH, mouse antiactin antibody, mouse antirabbit IgG antibody, and rabbit antimouse IgG antibodies were purchased from Santa Cruz Biotechnology (Santa Cruz, California). All other chemicals and reagents were purchased from Sigma unless otherwise specified.

### 2.2 Cell culture

The SCC25 and Ca9–22 human oral squamous carcinoma cell line was purchased from ATCC (Rockville, Maryland). YD10B OSCC cells were a gift from the Department of Oral Pathology, College of Dentistry, Yonsei University (Seoul, Korea). Cells were maintained at 37°C in a humidified atmosphere containing with 5% CO_2_ in Dulbecco's Modified Eagle Medium: Nutrient Mixture F‐12 (DMEM F‐12) and MEM/EBSS with 4 mM l‐glutamine, 1.5 g/L sodium bicarbonate, 4.5 g/L glucose, and 1.0 mM sodium pyruvate supplemented with 10% FBS (GIBCO‐BRL, Rockville, Maryland).

### 2.3 Treatment of embelin

Stock solutions of the Embelin (25 mM) which were made by dissolving them in DMSO were kept frozen at −20°C until use. The stock was diluted to their concentration with MEM/EBSS when needed. Prior to Embelin treatment cells were grown to about 80% confluence and then exposed to Embelin at different concentrations (2.5–300 μM) for 24 h. Cells grown in medium containing an equivalent amount of DMSO without Embelin served as control. For autophagy control, cells were grown in Earle's Balanced Salt Solution (EBSS).

### 2.4 MTT assay

Cells were placed in a 96‐well plate and were incubated for 24 h. Then they were treated with various doses of Embelin (2.5–300 μM) for 24 h. After cells were treated with 500 μg/mL of thiazolyl blue tetrazolium bromide (MTT solution), they were incubated at 37°C with 5% CO_2_ for 4 h. The medium was aspirated and formed formazan crystals were dissolved in DMSO. Cell viability was measured by an ELISA reader (Tecan, Männedorf, Switzerland) at 570 nm excitatory emission wavelength.

### 2.5 Hoechst staining

After embelin treatment, cells were harvested and cytocentrifuged onto a clean, fat‐free glass slide with a cytocentrifuge. Cells were stained in 4 μg/mL Hoechst 33342 for 10 min at 37°C in the dark and washed twice in PBS. The slides were mounted with glycerol. The samples were observed and photographed under an epifluorescence microscope (Carl Zeiss, Göettingen, Germany). The number of cells that showed condensed or fragmented nuclei was determined by a blinded observer from a random sampling of 3 × 10^2^ cells per experiment. Three independent experiments were conducted.

### 2.6 Flow cytometer analysis

For quantification of DNA hypoploidy, cells were harvested by trypsinization, and ice cold 95% ethanol with 0.5% Tween 20 was added to the cell suspensions to a final concentration of 70% ethanol. Fixed cells were pelleted, and washed in 1% bovine serum albumin (BSA)‐PBS solution. Cells were resuspended in 1 mL PBS containing 20 μg/mL RNase A, incubated at 4°C for 30 min, washed once with BSA‐PBS, and resuspended in PI solution (10 μg/mL). After cells were incubated at 4°C for 5 min in the dark, DNA content were measured on a CYTOMICS FC500 flow cytometry system (Beckman Coulter, FL, California) and data was analyzed using the Multicycle software which allowed a simultaneous estimation of cell‐cycle parameters and apoptosis. To quantify the development of acidic vesicular organelles (AVOs), the cells were stained with acridine orange (1 μg/mL) for 15 min, removed from the plate with trypsin‐EDTA, and analyzed using a FACScan flow cytometer. For autophagy inhibition, cells were pretreated with 1 mM 3‐MA for 1 h and incubated with Embelin for 24 h.

### 2.7 Fluorescence microscopy

Cells were grown on coverslips and treated with Embelin. After 24 h, cells were stained with 0.05 mM MDC, a selective fluorescent marker for autophagic vacuoles, at 37°C for 1 h. The cellular fluorescence changes were observed using a fluorescence microscope (Axioskop, Carl Zeiss, Germany). As an autophagy control, cells were starved using EBSS. For further detection of the acidic cellular compartment, we used acridine orange, which emits bright red fluorescence in acidic vesicles but fluorescence green in the cytoplasm and nucleus. Cells were stained with 1 μg/mL acridine orange for 15 min and washed with PBS. AVOs formation was obtained under a confocal microscope LSM 700 (Carl Zeiss, Germany).

### 2.8 Western blot analysis

Cells (2 × 10^6^) were washed twice in ice‐cold PBS, resuspended in 200 μL ice‐cold solubilizing buffer [300 mM NaCl, 50 mM Tris‐Cl (pH 7.6), 0.5% Triton X‐100, 2 mM PMSF, 2 μL/mL aprotinin, and 2 μL/mL leupeptin] and incubated at 4°C for 30 min. The lysates were centrifuged at 14 000 revolutions per min for 15 min at 4°C. Protein concentrations of cell lysates were determined with Bradford protein assay (Bio‐Rad, Richmond, California) and 20 μg of proteins were resolved by 12.5% SDS/PAGE. The gels were transferred to polyvinylidene fluoride (PVDF) membranes (Millipore, Billerica, Massachusetts and Amersham GE Healthcare, Little Chalfont, UK) and reacted with appropriate primary antibodies. Immunostaining with secondary antibodies was detected using SuperSignal West Femto (Pierce, Rockford, Illinois) enhanced chemiluminescence substrate and detected with Alpha Imager HP (Alpha Innotech, Santa Clara).

### 2.9 Statistical analysis

Statistical analysis data were expressed ± SD from at least three independent experiments. Statistical analyses used GraphPad Prism version 5.0 for Windows (GraphPad Software, San Diego, California). A one‐way ANOVA was used for Dunnett's multiple‐comparison test in the statistical analysis.

## 3 RESULTS

### 3.1 Toxicity and the apoptotic effect of embelin

**Figure 1:**
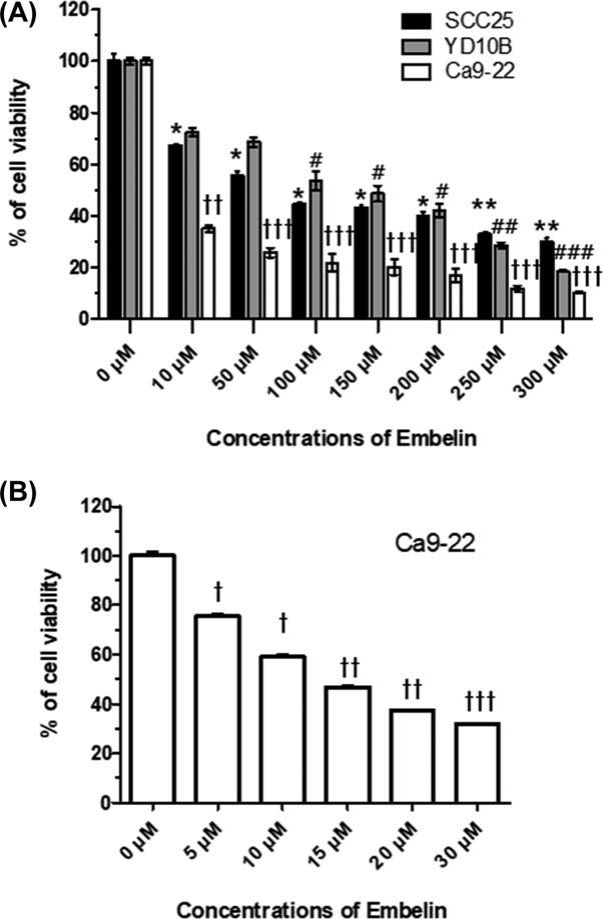
Effect of Embelin treatment on viability of OSCC cells. A, Embelin (10–300 μM) were to treat with three types of the OSCC cells (SCC25, YD10B, and Ca9–22) for 24 h. B, Ca9–22 cell were treated with Embelin (5–30 μM) and cell viability was analyzed using the MTT assay. The data were calculated as percent of vehicle control and expressed as the mean of at least three experiments. Data were expressed as the mean ± SD (*n* = 6) and analyzed by one‐way ANOVA using Dunnett's multiple‐comparison test (*P < .05, **P < .01, ***P < .001 on SCC25; #*P <* .05, ##*P <* .01, ###*P <* .001 on YD10B; *P <* .05, *P <* .01, *P <* .001 on Ca9–22 for the difference between the control and treatment groups)

**Figure 2:**
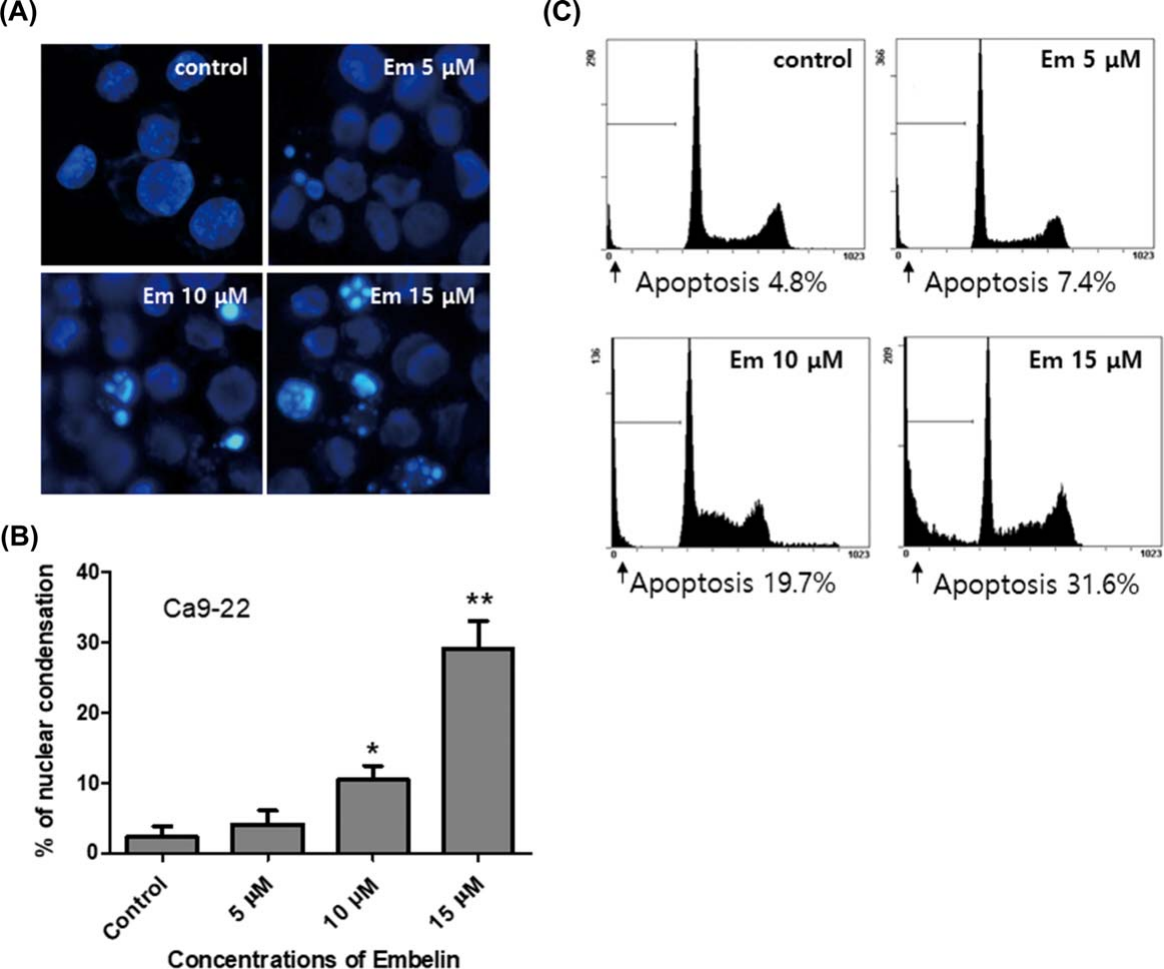
Embelin increased apoptotic cell death in Ca9–22 cells. A and B, Cells were treated with vehicle, Embelin (5–15 μM) for 24 h. C, The ratio of apoptotic cells was determined by flow cytometry analysis. Data were expressed as the mean ± SD (*n* = 3) and analyzed by one‐way ANOVA using Dunnett's multiple‐comparison test (*P < .05, **P < .01, for the difference between the control and treatment groups). [Color figure can be viewed at http://wileyonlinelibrary.com]

**Figure 3:**
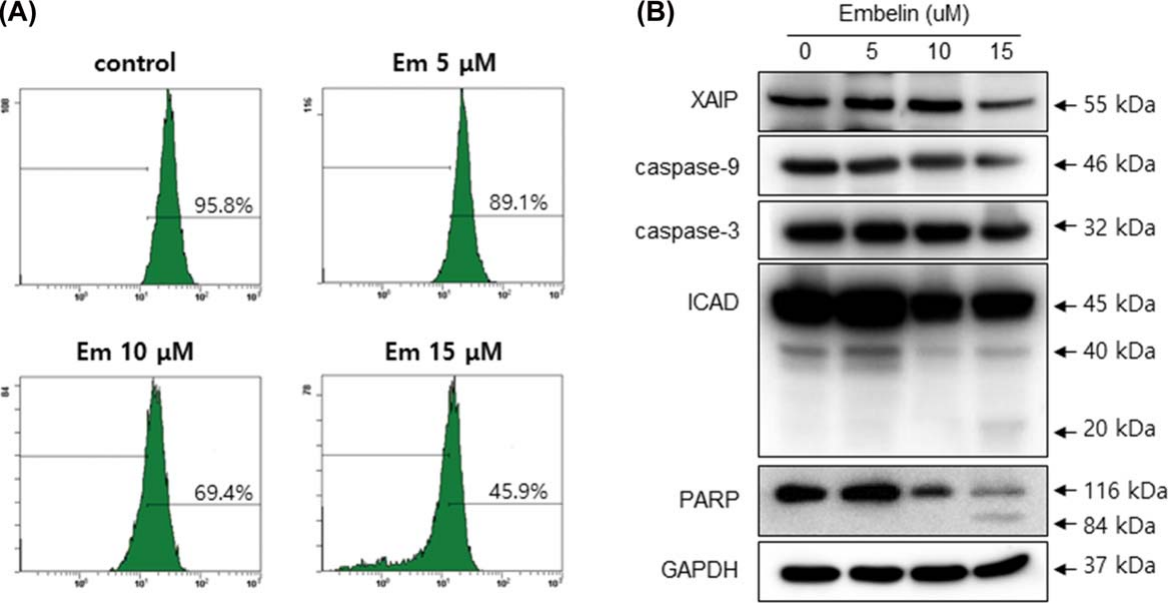
Embelin reduced mitochondrial membrane potential (MMP) and changed expressions of apoptosis‐related proteins. Cells were treated with Embelin for the indicated dose points, DiOC6 stained cells were measured by examining MMP by flow cytometry (A) and levels of XIAP, caspase‐9, caspase‐3, ICAD, and PARP were measured by Western blot analysis (B). [Color figure can be viewed at http://wileyonlinelibrary.com]

The effect of Embelin on OSCC (SCC25, YD10B, and Ca9–22) cells was investigated over a wide concentration range. Cells were treated with Embelin (0–300 μM) for 24 h, and then cell viability was assessed using the MTT assay. Embelin concentrations from 0 to 300 μM potently induced OSCC cell death. Thus, the viability of OSCC cells was decreased in a dose‐dependent manner by Embelin treatment. Also, our results showed decreased viability in the Ca9–22 cell lines, whereas SCC25 and YD10B cells were relatively resistant to the same doses (Figure 1A,B). Treatment with Embelin resulted in morphological and biochemical changes associated with apoptosis. Hoechst staining demonstrated that Embelin induced a change in nuclear morphology. The untreated Ca9–22 control cells had typical round nuclei, whereas the Ca9–22 cells treated with 5 to 15 μM Ca9–22 for 24 h displayed condensed and fragmented nuclei (Figure 2A) and an increased nuclear condensation ratio (Figure 2B). A flow cytometry assay was undertaken to test whether this cell death induction was mediated via apoptosis. The percentages of subdiploid cells, which are indicative of apoptotic cells, were increased in a dose‐dependent fashion by the Embelin treatment (Figure 2C). We further investigated the mechanism of Embelin‐induced cell death by examining the mitochondrial membrane potential (MMP) and apoptosis‐related proteins in Ca9–22 cells. As shown in Figure 3, Embelin reduced MMP and induced the degradation of caspase‐9, caspase‐3, and ICAD, and it also produced a processed ICAD 20 kDa and PARP 85 kDa fragments dose‐dependently.

### 3.2 Embelin treatment led to the induction of autophagy in Ca9–22 cells

**Figure 4:**
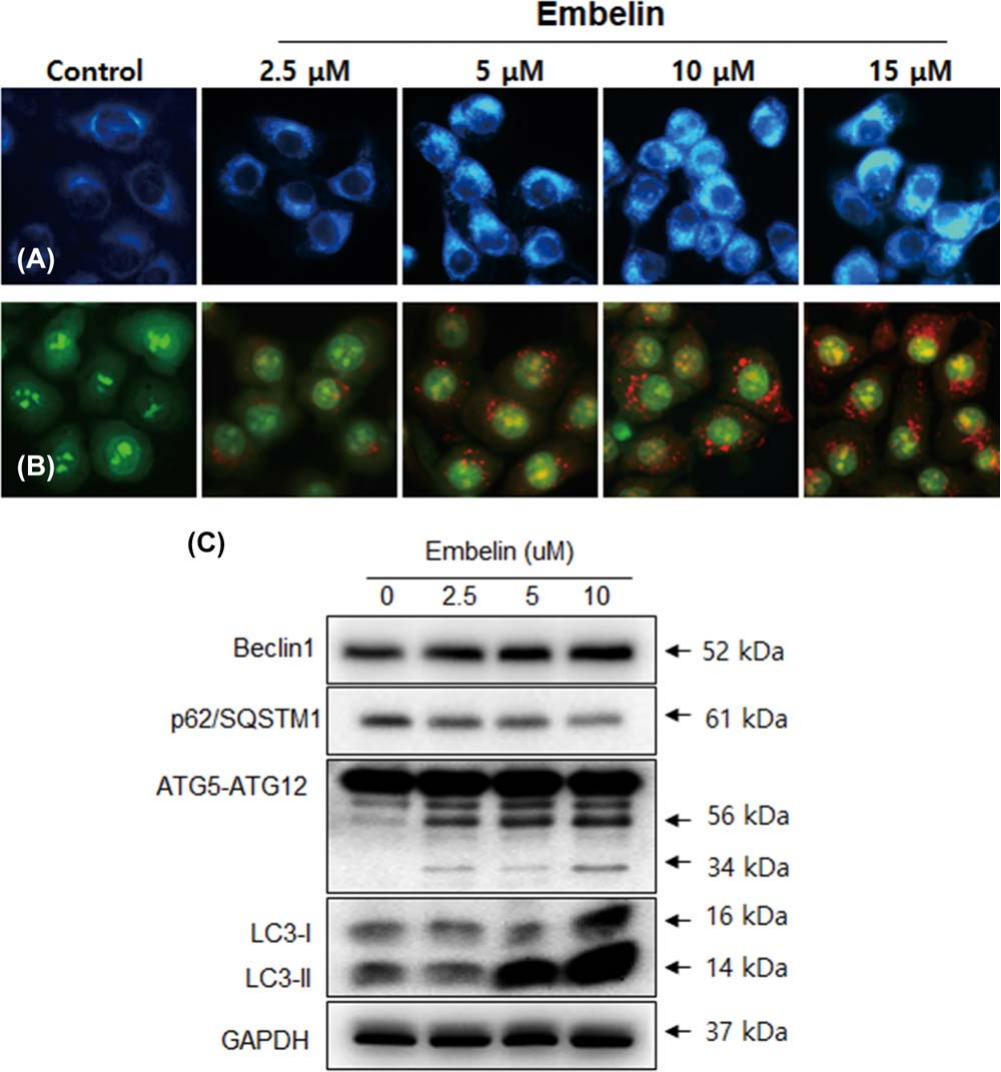
Embelin induced autophagy in Ca9–22 cells. Cells were stained with MDC (A) and acridine orange (B) as described in Materials and Methods. Ca9–22 cells were grown on coverslips and treated with Embelin (2.5–15 μM). Autophagic vacuoles were observed and imaged on a fluorescence microscope. C, Cells were treated with various concentrations of Embelin for 24 h and the expression levels of autophagy‐related proteins, such as Beclin‐1, p62/SQSTM1, ATG5‐ATG12, and LC3, were analyzed by Western blotting. [Color figure can be viewed at http://wileyonlinelibrary.com]

We next investigated whether autophagy occurred in Embelin‐treated Ca9–22 cells. When cells were stained with monodansylcadaverine (MDC), a selective fluorescent marker of autophagic vesicles, Embelin‐treated Ca9–22 cells exhibited strong staining when compared to control cells (Figure 4A). We confirmed the formation of autophagic vacuoles in response to Embelin by staining acidic vesicular organelles (AVOs) with acridine orange (AO). The AVOs, which represent autophagic vacuoles, were apparently formed in Ca9–22 cells (red fluorescence). As shown in Figure 4B, orange‐colored autophagic vacuoles were observed following treatment with 2.5 to 15 μM Embelin for 24 h. These findings indicated that Embelin treatment of Ca9–22 cells was sufficient to instigate an autophagic response as reflected by MDC and AO staining of autophagic vacuoles. This study tested whether Embelin induced autophagy in Ca9–22 cells by observing various autophagy markers, such as Beclin‐1, p62/SQSTM1, LC3, and ATG5–12 complex. Conversion rates of LC3‐I to LC3‐II as well as the total levels of LC3 proteins were increased in Embelin‐treated Ca9–22 cells in a dose‐dependent manner. The level of p62/SQSTM1, a protein that is activated by autophagy, was reduced in Embelin‐treated Ca9–22 cells. After treatment with Embelin, full length Beclin‐1 accumulated in a dose‐dependent manner (Figure 4C).

### 3.3 3‐Methyladenine blocked autophagy and promoted apoptosis by embelin

**Figure 5:**
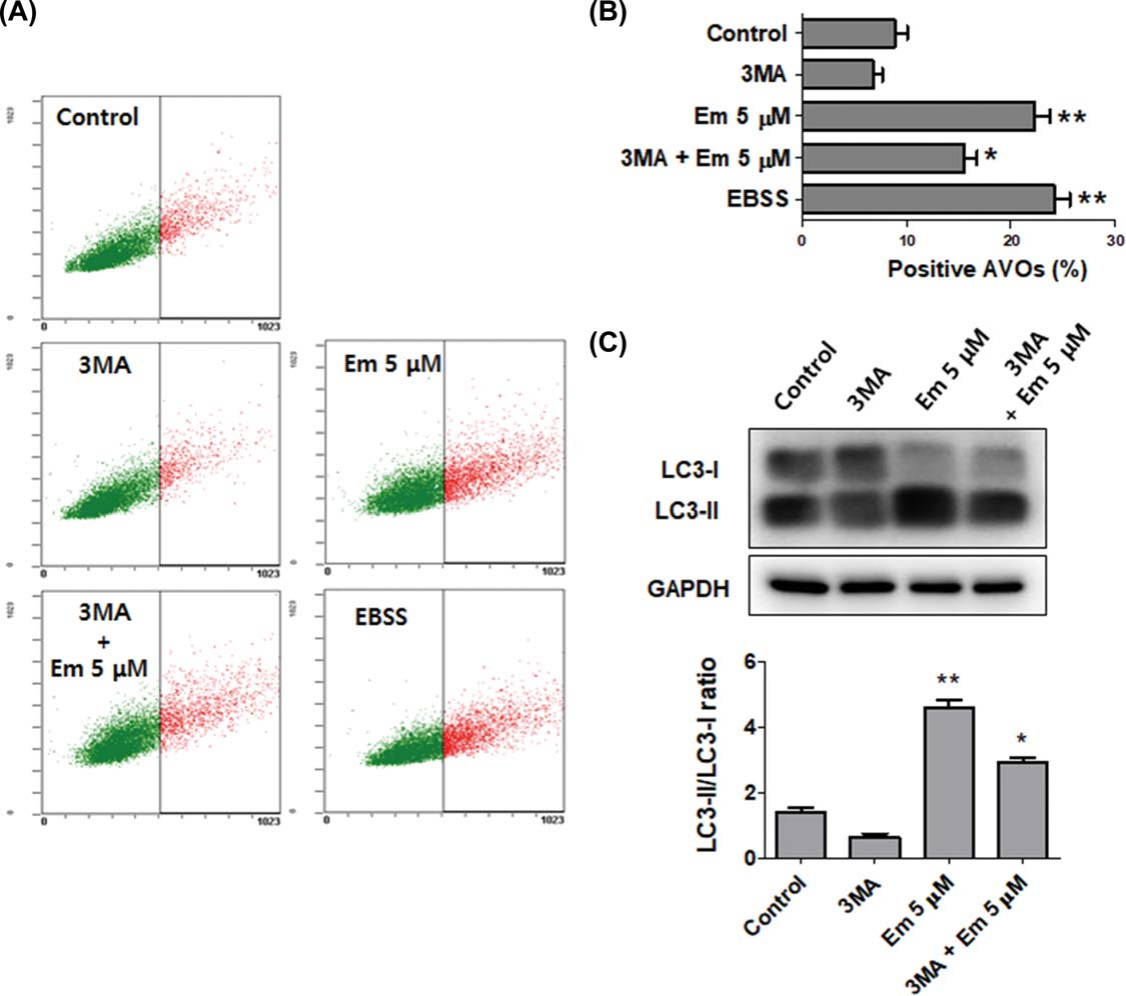
Embelin‐induced autophagy was inhibited by 3‐MA in Ca9–22 cells. Cells were pretreated with 1 mM 3‐MA for 1 h, and then exposed to 5 μM Embelin for 24 h. A and B, Vital staining was then performed using acridine orange, it is observed with the ratio red fluorescence quantified by flow cytometry. C, The expression levels of autophagy‐related protein LC3. As an autophagy control, cells were cultured in EBSS for 6 h. Data were expressed as the mean ± SD (*n* = 3) and analyzed by one‐way ANOVA using Dunnett's multiple‐comparison test (*P < .05, **P < .01 for the difference between the control and treatment groups). [Color figure can be viewed at http://wileyonlinelibrary.com]

**Figure 6:**
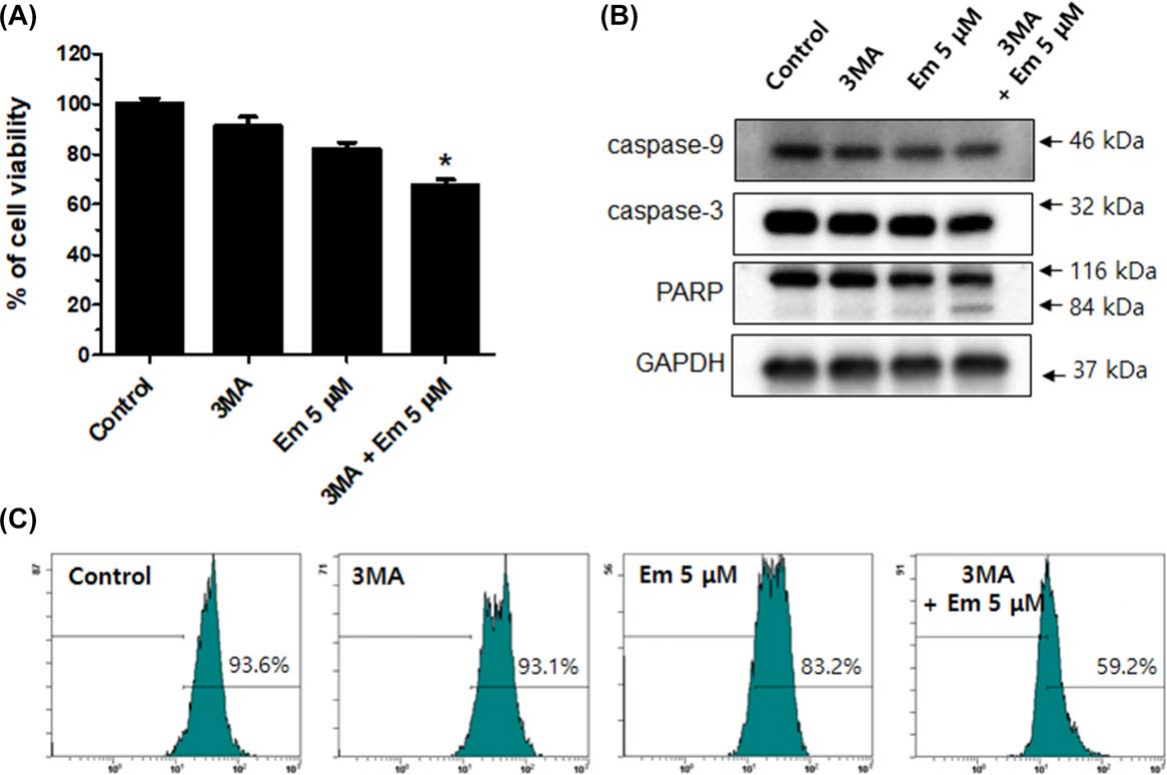
Embelin‐induced autophagy was involved apoptosis. Cells were pretreated with 1 mM 3‐MA for 1 h, and then exposed to 5 μM Embelin for 24 h. Cells were analyzed using the MTT (A), Western blot (B), and MMP (C). 3‐MA and Embelin combined treatment group showed down‐regulation of the cell viability, procaspase 9, 3, PARP, and MMP. Data were expressed as the mean ± SD (*n* = 3) and analyzed by one‐way ANOVA using Dunnett's multiple‐comparison test (*P < .05 for the difference between the control and treatment groups). [Color figure can be viewed at http://wileyonlinelibrary.com]

We further clarified the role of Embelin‐induced autophagy in Ca9–22 by investigating the effects of treatment with 3‐methyladenine (3‐MA), a selective autophagy inhibitor, on the Embelin‐treated Ca9–22 cells. The AVOs accumulated in 5 μM Embelin‐treated Ca9–22 cells that had been pretreated with 3‐MA. The 3‐MA prevented the formation of autophagic vacuoles induced by Embelin treatment. In addition, inhibitory effects of AVO formation by 3‐MA were confirmed by quantitatively measuring the red‐to‐green fluorescence ratio after AO staining (Figure 5A,B). A combined treatment with both 3MA and Embelin 5 μM showed that the conversion rate of LC3‐I to LC3‐II was decreased compared to a single treatment of Embelin on Ca9–22 cells (Figure 5C). We also intended to determine whether 3‐MA could promote Embelin‐induced apoptosis by assessing cell viability. The 1‐mM 3‐MA pretreatment and the Embelin‐treated group showed a greater decrease in cell viability than the group that received a single Embelin treatment (Figure 6A). Next, to find out whether 3MA could promote apoptosis, our results were verified by MMP and apoptosis‐related proteins. The group that was given a combination of 3‐MA and Embelin exhibited down‐regulation of the procaspase 9, 3, PARP, and MMP, and PARP cleaved form was shown (Figure 6B,C). The inhibition of the autophagic process with 3‐MA resulted in augmentation of the cytotoxic activity of Embelin. These results suggest that autophagy may be at least one of the pathways by which Embelin induces cell survival in Ca9–22 cells.

## 4 DISCUSSION

Autophagy is widely known as an important process in cell physiology for both cell survival and death.^19^ Autophagy begins with the elimination of cytoplasmic organelles in a double‐membrane vacuole, an autophagosome, which delivers them to a degradative organelle, the vacuole/lysosome, for breakdown and eventual recycling of the resulting macromolecules. Because numerous recent studies have shown that increased autophagic activity is associated with cell death,^12^, ^20^ autophagy is now considered to be a type of cell death. Embelin is an active ingredient in traditional herbal medicine used to treat inflammation and cancer^21^; many studies have shown that Embelin has a cytotoxic effect and inhibits cell proliferation in various cancer cell types,^6^, ^22^, ^23^, ^24^ presumably by activating the cell's apoptosis machinery.^5^, ^23^, ^25^ However, no reports to date have examined the relevance of apoptosis and autophagy for human oral cancer treatment.

Previous studies in our laboratory have also revealed that Embelin induced the cell death of Ca9–22 cells via apoptosis. The effect of Embelin on autophagic processes in Ca9–22 has not yet been determined. Our data demonstrated that the Embelin‐treated Ca9–22 cells had decreased viability and were undergoing cell death via apoptosis (Figures 1, 2, 3). We confirmed the autophagic effect of Embelin in Ca9–22 cells using AO and MDC staining. Embelin induced the formation of cytoplasmic vacuoles and acidic organelles (AVOs) in Ca9–22 cells (Figure 4A,B).

We also analyzed Embelin‐treated Ca9–22 cells for the presence of biochemical markers including p62/SQSTM1, LC3, ATG5‐ATG12 complex, and Beclin‐1, which are associated with autophagy. Embelin treatment directly affected the conversion of LC3‐II, the degradation of p62/SQSTM1 and full‐length Beclin‐1, and cleavage formation of ATG5‐ATG12 complex and Beline‐1 (Figure 4C). Several previous studies have reported that a relationship may exist between LC3 and p62/SQSTM1, and p62/SQSTM1 has been shown to selectively decrease in cells undergoing autophagy.^26^, ^27^, ^28^, ^29^ The process of autophagy mediates a nonspecific bulk degradation pathway that is responsible for the destruction of the majority of long‐lived proteins and some organelles. ATG12‐ATG5 conjugation systems are necessary for the formation of the autophagosome.^30^ Beclin‐1 (Bcl‐2‐interacting protein‐1) is a key protein in autophagy signaling, and it works in conjunction with Vps34, UVRAG, AMBRA‐1, and Barkor to assemble the PI3KC3 complex during the initiation of autophagosome formation.^31^, ^32^, ^33^ Several recent studies using different cell types and stimuli also reported that the caspase‐mediated cleavage of Beclin‐1 and ATG proteins enhances apoptosis.^34^, ^35^, ^36^, ^37^ Our results showed that Embelin led to the degradation of caspase‐9 and caspase‐3, and it assumes a decisive role on Beclin‐1 (Figure 3). To further clarify the role of autophagy in Ca9–22, our study demonstrated that Embelin‐induced cell death was increased by 3‐MA, an inhibitor of autophagy. Combination treatment with 3MA and Embelin showed various evidences of cell death as down‐regulation of cell survival rate, mitochondrial membrane potential, and cell death related proteins. This result suggests that Embelin‐induced autophagy is a prosurvival signal, it could act as an obstructive factor in oral cancer prevention. (Figure 6).

This is the first report of Embelin‐induced apoptosis and autophagy in Ca9–22 cells. As Embelin clearly can induce autophagy and is involved in the survival of Ca9–22 cells, the combination of Embelin and an effective autophagy inhibitor could be a potentially useful therapy for oral cancer treatment. The identification of the molecular mechanism by which Embelin acts will provide useful information for its development as a novel therapeutic agent for the management of Ca9–22.

## 5 ACKNOWLEDGEMENTS

This work was supported by the National Research Foundation of Korea(NRF) grant funded by the Korea government (No. NRF‐2017R1C1B5018034).

## ORCID

*In‐Ryoung KIm* http://orcid.org/0000-0003-0232-0385
